# Better Science Enough? Autoethnography of a Collaboration to Measure Structural Racism

**DOI:** 10.1093/geroni/igaf122.1476

**Published:** 2025-12-31

**Authors:** Paris Adkins-Jackson, Emily Haozous, Boeun Kim, Alicia Cooke, Melissa Hladek, Laura Samuel, Deidra Crews, Sarah Szanton

**Affiliations:** Mailman School of Public Health, Columbia University, New York, New York, United States; Pacific Institute for Research and Evaluation, Beltsville, Maryland, United States; University of Iowa, Iowa City, Iowa, United States; Johns Hopkins University, Baltimore, Maryland, United States; Johns Hopkins University, Baltimore, Maryland, United States; Johns Hopkins University, Baltimore, Maryland, United States; Johns Hopkins University, Baltimore, Maryland, United States; Johns Hopkins University, Baltimore, Maryland, United States

## Abstract

Addressing structural racism remains a critical challenge in health and social sciences, despite significant methodological advances over recent decades. This autoethnographic study explores a multidisciplinary collaboration’s approach to measuring structural racism through an intentional anti-racist praxis. Comprising biostatisticians, nurse scientists, epidemiologists, gerontologists, social scientists, and a nephrologist from diverse racial backgrounds, multi-career stages, and institutions, the research team employed innovative methodological approaches to examine structural racism’s complex dimensions. Our collaborative efforts included conducting life course history interviews with older adults racialized as Black, developing an infrastructure for structural racism measurement, organizing an interdisciplinary symposium, and producing a multi-season podcast exploring varied perspectives on racism measurement. Key themes emerging from our work centered on understanding structural racism through lived experiences, institutional infrastructures, and comprehensive measurement strategies. By critically reflecting on our collaborative process, we illuminate both the methodological challenges and potential transformative approaches to understanding and addressing systemic racialized inequities in health research.

